# Pathogenicity and virulence of Marburg virus

**DOI:** 10.1080/21505594.2022.2054760

**Published:** 2022-04-01

**Authors:** Mehedy Hasan Abir, Tanjilur Rahman, Ayan Das, Silvia Naznin Etu, Iqbal Hossain Nafiz, Ahmed Rakib, Saikat Mitra, Talha Bin Emran, Kuldeep Dhama, Ariful Islam, Abolghasem Siyadatpanah, Shafi Mahmud, Bonlgee Kim, Mohammad Mahmudul Hassan

**Affiliations:** a Faculty of Food Science and Technology, Chattogram Veterinary and Animal Sciences University, Chittagong, Bangladesh; b Department of Biochemistry and Molecular Biology, Faculty of Biological Sciences, University of Chittagong, Chittagong, Bangladesh; c Department of Genetic Engineering and Biotechnology, Faculty of Biological Sciences, University of Chittagong, Chittagong, Bangladesh; d Department of Pharmacy, Faculty of Biological Sciences, University of Chittagong, Chittagong, Bangladesh; e Department of Pharmacy, Faculty of Pharmacy, University of Dhaka, Dhaka, Bangladesh; f Department of Pharmacy, BGC Trust University Bangladesh, Chittagong, Bangladesh; g Division of Pathology, ICAR-Indian Veterinary Research Institute, Bareilly, India; h EcoHealth Alliance, New York, NY, USA; i Centre for Integrative Ecology, School of Life and Environmental Science, Deakin University, Victoria, Australia; j Ferdows School of Paramedical and Health, Birjand University of Medical Sciences, Birjand, Iran; k Genetic Engineering and Biotechnology, University of Rajshahi, Rajshahi, Bangladesh; l Department of Pathology, College of Korean Medicine, Kyung Hee University, Seoul, Korea; m Queensland Alliance for One Health Sciences, School of Veterinary Sciences, The University of Queensland, Gatton, Australia; n Department of Physiology, Biochemistry and Pharmacology, Faculty of Veterinary Medicine, Chattogram Veterinary and Animal Sciences University, Chattogram, Bangladesh

**Keywords:** Marburg virus, epidemiology, pathogenicity, transmission dynamics, cellular tropism, virulence

## Abstract

Marburg virus (MARV) has been a major concern since 1967, with two major outbreaks occurring in 1998 and 2004. Infection from MARV results in severe hemorrhagic fever, causing organ dysfunction and death. Exposure to fruit bats in caves and mines, and human-to-human transmission had major roles in the amplification of MARV outbreaks in African countries. The high fatality rate of up to 90% demands the broad study of MARV diseases (MVD) that correspond with MARV infection. Since large outbreaks are rare for MARV, clinical investigations are often inadequate for providing the substantial data necessary to determine the treatment of MARV disease. Therefore, an overall review may contribute to minimizing the limitations associated with future medical research and improve the clinical management of MVD. In this review, we sought to analyze and amalgamate significant information regarding MARV disease epidemics, pathophysiology, and management approaches to provide a better understanding of this deadly virus and the associated infection.

## Introduction

Marburg virus (MARV) causes deadly outbreaks with a high fatality rate. It is responsible for several outbreaks since its concurrent discovery and characterization in 1967 in Marburg, Germany; Frankfurt, Germany; and Belgrade, Yugoslavia (now Serbia). The majority of the MARV outbreaks occurred in Africa. MARV is into the NIAID Category A Priority Pathogen list, and is the primary cause of MARV disease (MVD) [1]. MVD is deadly and often becomes untreatable in humans and non-human primates (NHPs), resulting in hemorrhagic fever and organ dysfunctions, such as liver failure, the infection of the spleen, brain, and renal tissues, and coagulation problems throughout the body [2,3].

This virus belongs to the order *Mononegavirales*, the family *Filoviridae*, and the genus *Marburgvirus*. This genus only includes one species, named *Marburg marburgvirus*, generally known as Marburg virus [4]. Various studies have shown that MARV has five different lineages based on the phylogenetic analysis of genomic sequence data obtained from samples collected during different outbreaks [4,5]. These lineages have been reclassified into two separate viruses: the Ravn virus (RAVV) and MARV [4]. The human-to-human transmission characteristics of MARV are similar to those of the better characterized Ebolaviruses, including Ebola virus (EBOV) [6], Sudan virus, and Bundibugyo virus [7]. Due to the sporadic nature of MARV, distinguishing the natural reservoirs of this virus has been difficult. However, vigorous attempts and ongoing research have successfully determined the natural sources of this virus, which defines the viral transmission mechanism. The studies have substantiated that apart from *Rousettus aegyptiacus* bat species as the major natural source for MARV, *Hipposideros caffer* and some other Chiroptera can also serve as natural sources of infection [8,9]. Currently available clinical data have suggested that MVD has three stages associated with distinct symptoms [10]. Laboratory findings have indicated that the primary target of the virus is mononuclear phagocytic cells, followed by the epithelial cells in various organs [11]. However, diverse human exposure to this virus and the unorganized nature of currently available information have served as impediments to both researchers and policy-makers attempting to design appropriate guidelines for combating this disease. Though some drugs or vaccines have been successfully developed for MVD, its malignancy is still a great concern [12].

This review elaborately describes the history of MVD outbreaks, summarizes available information regarding the viral structure and genome, and describes the known sources of MARV and the transmission methods of both natural source-to-human and human-to-human infection pathways. This review also describes the pathophysiology, cellular tropism, immune evasion, and sites of major damage within the host body to provide a better understanding of the pathogenesis of MVD. Currently available clinical findings and management approaches are also described to help researchers to make appropriate decisions in preparation for future MVD outbreaks. No methods have been approved for the control of MVD outbreaks, and numerous studies remain necessary to develop drugs or vaccines. Our review will help future researchers comprehend the background of this virus and provide a profound understanding of the mechanism of infection, which will facilitate the MARV disease management and designing of future drugs and vaccines.

## Methodology

An organized literature search strategy was followed to find all the published articles, which reported outbreak history, genome sequence, structure, sources, pathophysiology, damaging prospects, cellular tropism, immune evasion, clinical findings, symptoms, transmission, and management of Marburg virus. To retrieve the information, we thoroughly searched for relevant literature through Google Scholar, Scopus, and PubMed between 1967 and October 2021. We have developed some specific Boolean words based on our objective, as shown in Table 1. These words were developed by using outcome term, descriptive term, population term, and area term. Boolean words “AND”, as well as “OR” along with [All fields] and [MeSH terms] searching techniques were used for literature searching in Scopus and PubMed. Advanced search strategy has also been maintained in Google Scholar, and some adjustments have been made on the basis of all search engine requirements.

**Table 1.** Electronic database search algorithm

Term	Boolean keywords
Outcome	Detection OR Identification OR Investigation OR Incident OR History OR Characterization OR Occurrence OR Rate OR Approaches OR Management OR Case OR Prospects OR Findings
Descriptive	Outbreak OR Fatality OR Transmission OR Genome OR Structure OR Damaging OR Dissemination OR Cellular tropism OR Immune evasion OR Pathophysiology OR Clinical OR Symptoms OR Sources
Population	Marburg Virus OR MARV OR Filovirus OR Filoviridae
Area	Africa OR DRC OR Congo OR Angola OR South Africa OR Zimbabwe OR Kenya OR Uganda OR Europe OR Russia OR Germany OR Netherlands OR America OR USA

## Genome and structure of Marburg virus

MARV is a pleomorphic virus which is observed in six, circular, U, rod-like and most commonly in filamentous shape [13]. Usually, MARV virions are of 80 nm in diameter and although their length varies greatly, the average length of a MARV virion is 790 nm [14]. The surface of the virion is shielded with 5 to 10 nm-long spikes placed at interludes of approximately 10 nm [13,15].

**Figure 1.**
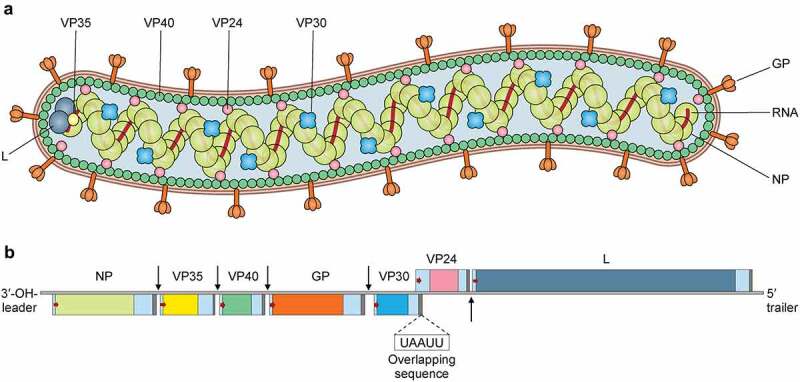
Virion structure and genome organization of Marburg virus. Top, the Marburg virus structure along with depicting the structural proteins. Bottom, an illustration of the genome organization of the Marburg virus. This seven-gene strain of Marburg virus has been drawn roughly to scale. The light blue boxes indicate noncoding areas, as well as the colored box code regions for genes. The red arrows demonstrate the position of the transcriptional start signals, whilst the pale brown bars highlight conserved transcriptional stop signals. The genes are segregated by intergenic regions, indicated using black arrows, with the exception of the overlapping sequence (black triangle) between VP24 and VP30. At the extreme ends, the 3' and 5' trailer sequence is shown.

It is a non-segmented negative-sense virus which contains a 19.1 kb long RNA genome that encodes seven genes in the following linear order-3'-NP-VP35-VP40-GP-VP30-VP24-L-5' [16]. Each of these seven genes has a highly conserved transcription start and stop signal and also possess unconventionally long noncoding nucleotide sequence at the 3' and 5' extremities [16]. These noncoding regions containing *cis*-acting element play role in replication, transcription and also in DNA packaging [16,17]. All but two genes of MARV are segregated by 4–97 nucleotides long intergenic regions, the transcription stop signal of VP24 and the transcription start signal of VP30 gene share a five nucleotide long overlapping sequence UAAUU [18] (Figure 1).

All of the seven genes in the MARV genome are monocistronic [16] and are responsible for encoding the following seven structural proteins- Nucleoprotein (NP), Viral protein 35 (VP35), Viral protein (VP40), Glycoprotein (GP), Viral protein 30 (VP30), Viral protein 24 (VP24) and Large Protein (L). The genome of MARV is encapsulated with a nucleocapsid complex made up of four structural proteins- NP, VP35, VP30 and L [19]. NP is the major nucleocapsid protein which forms a tubular helical structure which interacts with VP35 and the resulting complex interacts with L [19,20]. Here the L protein functions as the RNA dependent RNA polymerase and VP35 works as the polymerase cofactor [21]. All four nucleocapsid complex proteins play essential role in viral genome replication and transcription. MARV contains a host derived membrane layer, which is spiked in regular intervals. These spikes, made up of heavily glycosylated protein (GP), are necessary for attachment to susceptible host cells [15]. The inner matrix of the virion is formed by VP40 and is responsible for budding and association with matrix and nucleocapsid [22,23]. VP24 shows interaction with both NP and other cellular membranes and is associated with the release of progeny virions from the cell [24]. Table 2 illustrates the functions and characteristics of the proteins in MARV.

**Table 2.** Marburg virus genes, proteins and their characteristics and functions

Gene (sequence)	Gene/ORF length (nt)	Protein (abbreviation)	Amino acid	Attributes	Functions	Ref.
NP (1)	2796/2088	Nucleoprotein (NP)	695	Component of RNP complex; phosphorylated; binds to VP35, VP40, VP30, and VP24;creates helical polymer by homo-oligomerization; second most found protein virions.	Formation of NC and cellular inclusion body; Encapsidation of RNA genome as well as antigenome; Replication and transcription; Budding	[25–27]
VP35 (2)	1557/990	Viral protein 35 (VP35)	329	Components of RNP complex; homo‑oligomerizes; weakly phosphorylated; binds to dsRNA, NP, and L.	Formation of NC; RdRp cofactor; Replicase transcriptase cofactor; IFN antagonist;	[25,28–30]
VP40 (3)	1405/912	Viral Protein 40 (VP40)	303	Consists of two distinct functional modules; contains a late budding motif; homo‑oligomerizes to form dimers, circular hexamers, octamers; binds ssRNA, VP35; hydrophobic; membrane-associated; most common proteins in virions and infected cells	Matrix component: Negative regulator of transcription and replication; Budding and host adaption; regulation of the morphogenesis of the virion and egress; hinders JAK‑STAT pathway.	[25,31–33]
GP (4)	2846/2046	Glycoprotein (GP_1,2_)	681	Creates heterodimers using GP_1_ and GP_2_ subunits; mature protein is found as a trimer of GP1,2 heterodimers; ability to insert into membranes; Acylated, prominently N- and O‑glycosylated, phosphorylated; GP_1,2_ turns into soluble GP_1,2Δ_ by ADAM17; Class I fusion and type I transmembrane protein	Attachment of virions to susceptible cells using cellular attachment factor: determination of cell and tissue tropism; Receptor binding; induction of virus‑cell membrane; Tetherin antagonist; Function of GP_1,2Δ_ is yet to be known.	[25,34]
VP30 (5)	1249/846	Viral protein 30 activator (VP30)	281	Components of RNP complex; Highly phosphorylated; Contains a Zinc binding domain, and binds to ssRNA, NP and L	Formation of NC; Initiation, reinitiation and antitermination and enhancement of transcription.	[35,36]
VP24 (6)	1287/762	Viral protein 24 (VP24)	253	Components of RNP complex; Membrane-associated;Homo-tetramerizes;Hydrophobic	Formation and maturation of NC; Negative regulation of transcription; regulation of replication; virion morphogenesis regulatory function; regulation of viral egress; Activation of cytoprotective responses.	[25,37–40]
L (7)	7745/6996	Large protein (L)	2331	Components of RNP complex; Homodimerizes; Binds to VP35, VP30, genomic and antigenomic RNA; mRNA capping enzymes.	Catalytic domain of RdRp; Replication of genome; Transcription of mRNA	[41,42]

Gene length and ORF length are collected from GenBank accession numbers NC_001608 (MARV). (RNP- Ribonucleoprotein; NC- nucleocapsid; dsRNA- double stranded RNA; RdRp- RNA-dependent RNA polymerase; IFN- interferon; ssRNA- single stranded RNA; JAK‑STAT- Janus kinase-signal transducer and activator of transcription; ADAM17- ADAM Metallopeptidase Domain 17).

## Historical outbreaks and disease epidemiology

The first outbreak of MARV was documented in Marburg, Germany, in 1967, where scientists and laboratory technicians were doing experiments with tissue derived from African green monkeys (*Chlorocebus aethiops*), collected from Uganda in an attempt to produce vaccines for polio [43,44]. Concurrently, additional outbreaks erupted in Yugoslavia and Frankfurt. Electron microscopy was performed to identify and characterize the virus on plasma from infected guinea pigs, and it was named the “Marburg virus” [44].

The next documented MARV outbreak occurred in South Africa in 1975, which infected three people. The first individual became infected while traveling to Zimbabwe, and his companion and a nurse became infected through human-to-human transmission [45,46]. In 1980, a third outbreak of MARV was identified in Kenya, where a male became infected following a visit to Kitum cave, and a doctor became infected while treating this individual [47]. Another small outbreak occurred in Kenya in 1987, which resulted in the detection of a new strain of MARV. However, the only infected individual was a 15-years-old Danish boy who became infected 7 days after a visit to Kitum cave [48].

In 1988, 1990, 1991, and 1995, laboratory accidents resulted in occurrences of MARV infection in Russia (Table 3). The subsequent MARV outbreak took place between 1998 and 2000, in the DRC (Democratic Republic of the Congo), which was associated with 154 total infections. Initially, young gold miners became infected during their mining work in the village of Durba, and the outbreak later spread to the nearby village of Watsa. At least nine genetically diverse MARV lineages were identified during this outbreak [8,49]. Another severe MARV outbreak took place in Angola’s Uige region, which was first identified in October 2004, and continued through July 2005. The outbreak was initially identified as due to the death of a hospital employee in Uige, and continued to spread through other provinces. The largest numbers of infections and deaths associated with a single outbreak to date occurred during this outbreak, which resulted in 252 infection cases and 227 deaths, representing a fatality rate of 90%. Clinical investigations revealed similarities between the genetic sequences of previously identified MARV isolates and those associated with the 2004–2005 Angolan MARV outbreak [50].

**Table 3.** Marburg virus outbreaks epidemiology and case fatality rate (CFR) in accordance with the outbreak strain

Month & Year	Country	City	Strain(s)	Origin (some origins are precarious)	Number of cases	Deaths	CFR (Case-fatality rate)	Epidemiology	Ref.
August, 1967	Germany	Marburg	MARV Ci67, MARV Flak, MARV Hartz, MARV “L”, MARV Porton, MARV Popp, MARV Rat, MARV Voe	Uganda	24	5	20.83%	Infection from African green monkey’s infected tissues imported from Uganda	[59,60]
	Germany	Frankfurt			6	2	33.33%		
	Yugoslavia (Serbia)	Belgrade			2	0	0.00%		
February, 1975	South Africa	Johannesburg	MARV Cru, MARV Hogan, MARV Ozo	Zimbabwe	3	1	33.33%	Nearly uncertain, may be visiting to Chinhoyi caves	[46,61]
January, 1980	Kenya	Nairobi	MARV Mus	Kenya	2	1	50.00%	Working near Kitum Cave, Mount Elgon National Park	[47]
August, 1987	Kenya	Nairobi	RAVV Ravn, RAVV R1	Kenya	1	1	100.00%	Visiting Kitum Cave, Mount Elgon National Park	[48]
1988	Russia	Koltsovo	MARV “U”	Russia	1	1	100.00%	Unexpected event in laboratory	[62]
1990	Russia	Koltsovo	-	Russia	1	1	100%	Unexpected event in laboratory	[63]
1991	Russia	Koltsovo	MARV Popp	Russia	1	0	0.00%	Unexpected event in laboratory	[64]
1995	Russia	-	MARV Popp	-	1	0	0.00%	Unexpected event in laboratory	[65]
1998–2000	Democratic Republic of Congo	Durba, Watsa	Lots of strains	Durba, Democratic Republic of the Congo	154	128	83.12%	Infection developed during gold mining in Goroumbwa cave	[66]
2004–2005	Angola	Uige	MARV Angola	Uíge Province, Angola	252	227	90.08%	Uncertain	[7]
June, 2007	Uganda	Kamwenge	MARV-01Uga 2007, RAVV-02Uga 2007	Kamwenge District, Uganda	4	1	25.00%	Infection developed during gold mining in Kitaka Cave	[10]
January, 2008	USA	Colorado, City unreported	-	Uganda	1	0	0.00%	Visiting a Python Cave in Maramagambo Forest	[67]
July, 2008	The Netherlands	Leiden	MARV Leiden	Uganda	1	1	100.00%	Visiting a Python Cave in Maramagambo Forest	[11]
October, 2012	Uganda	Kabale, Ibanda, and Kamwenge	-	-	15	4	26.67%	-	[46,47]
October, 2014	Uganda	Kampala	-	-	1	1	100.00%	Infection probably spread from Mengo Hospital	[48]
October, 2017	Uganda	Kween	-	-	4	3	75.00%	Infections probably spread from a cave in Kaptum grazing ground or a cave on the slope of Mount Elgon	[56]
August, 2021-September, 2021	Guinea	Gueckedou	-	-	1	1	100.00%	-	[57,58]

**Figure 2.**
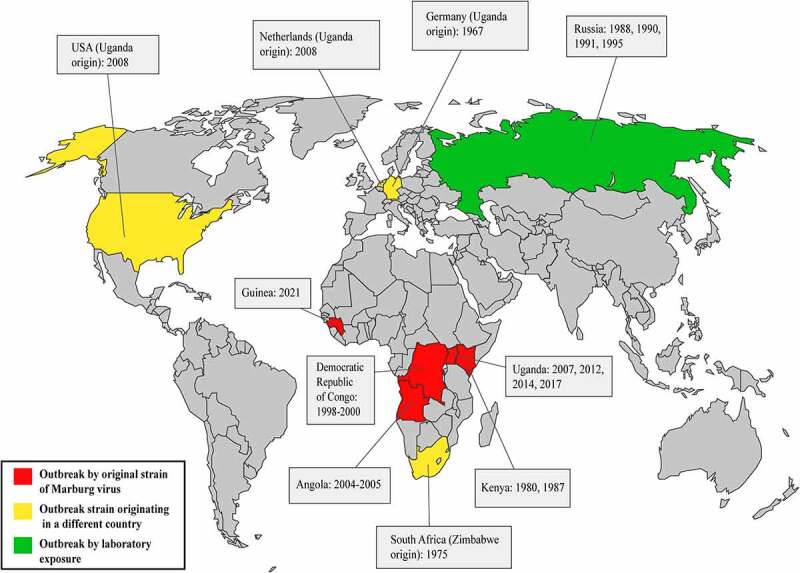
Outbreak history of Marburg virus. The red color on the map demonstrates the occurrences of outbreaks associated with new strains of the Marburg virus. Primarily, MARV outbreaks have been identified in four countries in Africa: The Democratic Republic of Congo, Angola, Kenya, and Uganda. However, an outbreak in Zimbabwe was also associated with a novel strain of the Marburg virus, which caused an outbreak in South Africa. The yellow color on the map shows infections in which the source is known to have originated from another country. This type of outbreak occurred in Germany, the Netherlands, the USA, and South Africa. The green color shows the outbreaks associated with unintentional laboratory exposures. This sort of epidemic took place only in Russia.

To date, no other severe outbreaks have occurred following the 2004–2005 Angolan outbreak, apart from certain sporadic cases in various zones of the world. Four miners were infected in the gold and lead mines of Uganda’s Kamansanga district during mining operations in 2007 [51]. This outbreak occurrence of Uganda related to the two following MARV cases, the first one was found in the USA and the next one was found in the Netherlands (Figure 2). However, the US patient survived this MARV infection, but the Dutch patient died afterward (Table 3). Interestingly, these two individuals became MARV infected while traveling Uganda’s Maramagambo forest python cave [52,53]. Moreover, Uganda was the site of the next three epidemics (Figure 2). The first one occurred in the Kabale region of Uganda in 2012, lasting for three weeks, during which fifteen individuals became MARV infected, four of whom died later on. The genomic sequence of 2012 Uganda’s outbreak strain was similar to the formerly identified strain’s genomic sequences [54]. The following MARV outbreak also took place in Uganda in 2014, in the city of Kampala, when a healthcare worker became infected and passed away after a few days. This MARV strain also had similarities in genome sequences with the formerly determined MARV strain’s genome sequences from Egyptian fruit bats [55]. Another recent epidemic occurrence of MARV took place in Uganda’s Kween region in 2017. In this outbreak, 4 people in the same family were infected with MARV and only one person survived later. This strain’s genome sequences also had similarities with the previously identified strain [56]; however, the clinical findings associated with this outbreak remain inadequate, and intense research is currently ongoing.

Lastly, the most recent outbreak has been declared in Guinea in August 2021 and ended in September 2021, where one male got infected and died; but there are no data available regarding the strain [57,58].

The disease epidemiology of MARV, along with its case fatality rate is shown in Table 3, which were collected from all outbreak data associated with MARV infection.

## Source and transmission of Marburg virus

**Figure 3.**
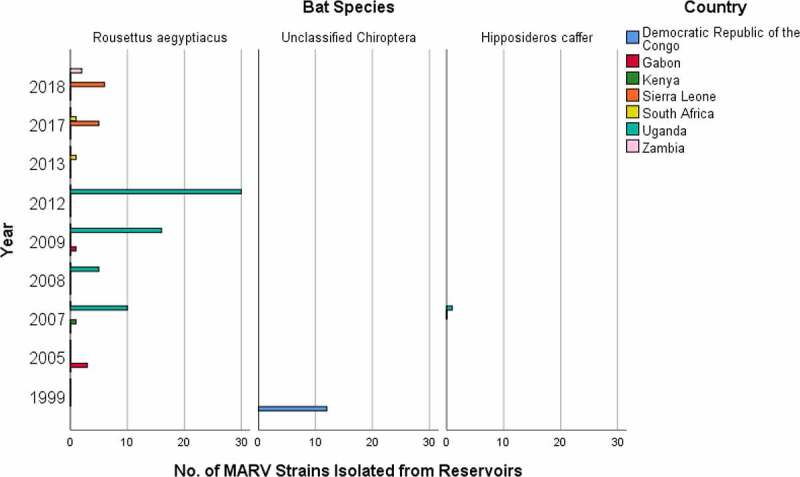
Number of MARV strains identified from reservoir bat species, which were responsible for the disease spread in different years and countries.

Historically, numerous MARV strains have been isolated from both animals and humans. Animals, specifically bats are the natural reservoirs of MARV [9]. MARV strains were isolated from reservoir hosts in different courses of time to comprehend their genomic variations and disease pathogenesis. Moreover, the presence of MARV in reservoir hosts have been substantiated through some laboratory tests, most specifically through PCR-positive tests. The number of isolated strains responsible for disease spread within the reservoir host species at different year and country is shown in Figure 3 (Supplementary Table S1). *Rousettus aegyptiacus* species of bat most frequently acts as a reservoir of MARV, along with *Hipposideros caffer* and some unclassified Chiroptera as the minor sources. It is because the majority of the MARV strains were collected from *Rousettus aegyptiacus* species, which included 3 from Gabon in 2005 [68]; 1 from Kenya [69] and 10 from Uganda in 2007 [8]; 5 from Uganda in 2008 [8,70]; 1 from Gabon [71] and 16 from Uganda in 2009 [70]; 30 from Uganda in 2012 [72]; 1 from South Africa in 2013; 5 from Sierra Leone [73] and 1 from South Africa in 2017 [74]; and 6 from Sierra Leone [73], and 2 from Zambia in 2018 [75]. Furthermore, 12 MARV strains were collected from unclassified Chiroptera in DRC in 1999 [9], and 1 MARV strain was collected from *Hipposideros caffer* in Uganda in 2007 [8].

**Figure 4.**
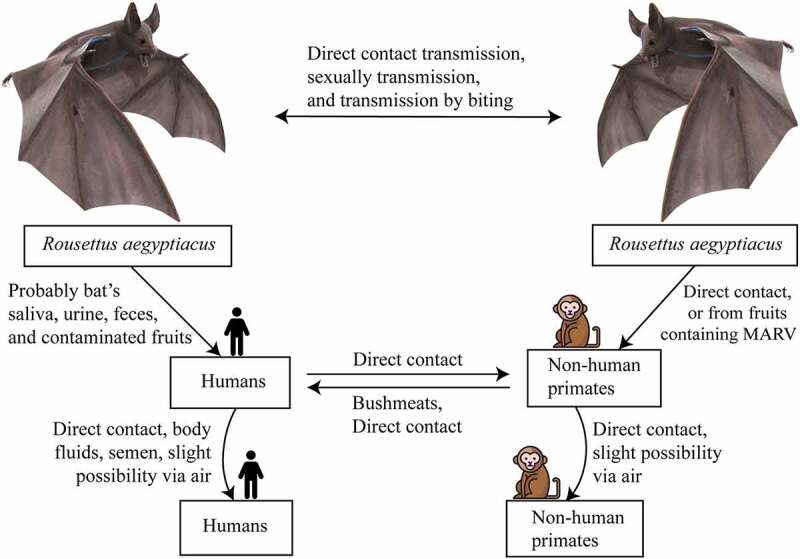
Transmission and spread of Marburg virus. Reservoirs of the Marburg virus, such as African fruit bats, can spread the virus among themselves by direct contamination, through sexual transmission, or due to biting. Direct contact with reservoir hosts or viral-contaminated fruit consumption may spread the virus to humans and non-human primates (NHPs). Transmission between humans and NHPs may occur through direct contact, and NHP-to-human transmission occurs due to bushmeat consumption and through direct contact. Direct contact and aerosols can facilitate both human-to-human and NHP-to-NHP transmission.

The bat-to-bat transmission of MARV strains may occur in several ways. A recent study that detected the shedding of MARV in rectal, oral, and urine samples from MARV-inoculated bats, also found that MARV is present in oral and blood samples of in-contact bats. This study proved the horizontal transmission of MARV from infected bats to the in-contact bats (Figure 4) [76]. The previous study found the presence of MARV in the lung, intestine, kidneys, bladder, salivary gland, and females’ reproductive tract tissues of inoculated bats, which helped to presume that MARV transmission may occur vertically or horizontally within the reservoirs [77]. It is also hypothesized that bat-to-bat transmission may occur through biting [7], sexual interactions [7,70], or by hematophagous arthropods [76].

Intermediate hosts, such as non-human primates (NHPs) and animals hunted for bushmeat, in addition to natural reservoirs, for example saliva, urine, and excrement of bat, are the primary vectors of MARV transmission. The transmitting paths from reservoir hosts to humans remain unknown [78,79]. However, it is possible that bat’s saliva, urine, and feces, as well as MARV-contaminated fruits, are the probable causes of transmission in humans as well as NHPs (Figure 4) [76,80]. Table 4 provides all of the known primary and secondary sources of MARV transmission, along with their infection pathways.

**Table 4.** Sources and infection types of Marburg virus

Primary infection		Secondary infection	
Primary Host/Natural Reservoir	Infection Medium	Secondary Host	Infection medium
Egyptian Fruit Bat (*Rousettus aegyptiacus*) [7,70,73]	Blood [77]	Non-Human Primates [81,82]	Blood [82,83]
	Urine [73,76]		Tissue [82,83]
	Stool [76]		Body fluids (e.g. semen) [84]
	Saliva[7,73,76]	Human [56,61]	Blood [85,86]
	Body tissue [73]		Tissue [85]
	Other body Fluids [76]		Dead-body [47,85,86]
			Body Fluids (e.g. saliva, stool, tears, urine, semen, breast-milk) [47,86]

In the early stages, MARV may spread to humans by infected intermediate animals. Besides, MARV may transmit due to sexual intercourse within humans, since the presence of virus antigens in the semen of infected males has already been substantiated [78]. The studies on EBOV also suggested the persistence of EBOV RNA as well as infectious EBOV in semen after the patients’ recoveries from Ebola virus disease, which shares a similar human-to-human transmission dynamic as MVD [87–89]. Furthermore, the virus can transmit from one human to another due to direct contact with blood, as well as other body fluids, for example feces, saliva, urine, teardrops, mucous, and breast milk (Figure 4). Managing and providing healthcare service to the MARV infected patient, and inappropriate handling of human corpses also leads to increased chances of MARV transmission [90]. Transmission has also been hypothesized to occur through the air during an outbreak, as the virus may survive aerosols [91], hence mucosal membranes are highly susceptible to viral transmission through the air (Figure 4). Apart from aerosol survival, an important study suggested that Lake Victoria MARV can also survive on liquids for longer periods, and on solids surfaces (plastics and glasses) for more than 3 weeks at low temperature [92]. Therefore, fomite transmission of MARV can play an important role in virus spreading, especially during an outbreak. Other forms of MARV transmission include the breakdown of skin cells, and the parenteral or enteral introduction of drugs and foods [93].

## Clinical findings and symptoms

Most available clinical data were obtained from a few large outbreaks, especially the 1967 outbreak of Germany and Belgrade of Yugoslavia, the 1998 to 2000 outbreak of DRC, and the 2004 to 2005 outbreak of Angola [44,60,94]. The clinical characteristics of a MARV-infected patient may vary depending upon various factors, including strain virulence, physical status, host susceptibility, and medical maintenance. To date, the reported incubation period has ranged between 2–21 days, and the average duration is 5 to 9 days, in humans [60,95].

MHF can be divided into 3 distinct phases; initial generalization phase, that is followed by early organ phase, and then the late organ phase or convalescence phase, according to disease progression [96] (Table 5).

**Table 5.** MVD symptoms, according to the phase of infection

Phase of Infection	Types of Symptoms	Diagnostic Symptoms
Generalization Phase (day 1–5)	General Symptoms	High fever (39-40° C)
		Severe headache
		Myalgia
		Chills
		Malaise
		Conjunctivitis
		Dysphasia
		Enanthem
		Pharyngitis
		Maculopapular rash
		Lymphadenopathy
		Leukopenia
		Thrombocytopenia
	Debilitating Symptoms	Fatigue
		Loss of appetite
		Abdominal pain
		Severe weight loss
		Severe nausea
		Vomiting
		Watery diarrhea
		Anorexia
Early Organ Phase (day 5–13)	General Symptoms	Conjunctival infection
		Prostration
		Dyspnea
		Exanthema
		Edema
		Abnormal vascular permeability
	Neurological Symptoms	Confusion
		Encephalitis
		Irritability
		Delirium
		Aggression
	Hemorrhagic Symptoms	Mucosal bleeding
		Melena
		Petechiae
		Bloody diarrhea
		Visceral hemorrhagic effusions
		Uncontrolled leakage from venipuncture sites
		Hematemesis
		Ecchymoses
Late Organ Phase/Convalescent Phase (day 13-20+)	General Symptoms	Severe metabolic disturbances
		Convulsions
		Severe dehydration
		Multiorgan disfunction
		Anuria
		Orchitis
		Myalgia
		Exhaustion
		Partial amnesia
		Sweating
		Peeled skin from rash areas
		Secondary infection
	Common complications during convalescent phase	Arthralgia
		Hepatitis
		Asthenia
		Ocular disease
		Psychosis

### Phase 1 (generalization phase)

The initial generalization phase lasts for five days after disease onset. The early symptoms that are present during this phase include generic flu-like characteristics, accompanied by high fever (39–40 °C). In addition, debilitating symptoms, including fatigue, loss of appetite, abdominal pain, severe weight loss, severe nausea, vomiting, watery diarrhea, and anorexia, have been reported by many patients. Severe headaches, myalgia, chills, and malaise are also common signs [46,83,93]. The end of this initial phase is frequently characterized by conjunctivitis, dysphasia, enanthem, and pharyngitis. A characteristic maculopapular rash may develop on different body parts (prominently on the neck, back, and stomach), which represents a distinctive characteristic of filovirus infection. Other symptoms include lymphadenopathy, leukopenia, and thrombocytopenia [97–101].

### Phase 2 (early organ phase)

A sustained high fever and other general symptoms accompany the early organ phase, which lasts from five to thirteen days after the onset of symptoms. Patients may manifest conjunctival infection, prostration, shortness of breath (dyspnea), viral exanthema, irregular vascular permeability, and edema [93]. Neurological symptoms, such as confusion, encephalitis, irritability, delirium, and aggression, have also been reported in patients [46,83,102]. Approximately 75% of patients are present with hemorrhagic manifestations, including mucosal bleeding, melena, petechiae, bloody diarrhea, visceral hemorrhagic effusions, uncontrolled leakage from venipuncture sites, hematemesis, and ecchymoses. Bleeding from the nose, gums, and vagina has also been reported. Because hemorrhagic symptoms are present in some patients, the MARV infection is typically denoted MHF in these cases, although this is still not favorable to all cases. Multiple organs, including the kidney, liver, and pancreas, are affected during this phase of infection. Elevated serum activity was also noticed in most infected individuals [98–101].

### Phase 3 (late organ phase/convalescent phase)

The late stage of MARV infection results in two distinct outcomes: infection either becomes fatal or patients enter a prolonged phase of restoration. Fatality generally occurs between eight and sixteen days after the onset of symptoms. Typically, shock and multi-organ failure are the primary drivers of death [83,102]. The late organ phase (in non-fatal cases) starts on day thirteen and lasts until day twenty and beyond during the course of the disease. Severe metabolic disturbances, including convulsions and severe dehydration, result in severe negative effects on overall patient health, resulting in multi-organ dysfunction and anuria. Orchitis has been reported in some cases during this phase. Neurological symptoms persist during this stage. An additional complication includes spontaneous abortion in pregnant women [66,102]. Myalgia, exhaustion, partial amnesia, sweating, peeling skin in rash-affected areas, and secondary infections are noticeable signs during this prolonged phase. Arthralgia, hepatitis, asthenia, ocular disease, and psychosis are common complications during the convalescent phase of the infection [98–101].

## Viral dissemination and cellular tropism of Marburg virus

### Viral entry and budding

**Figure 5.**
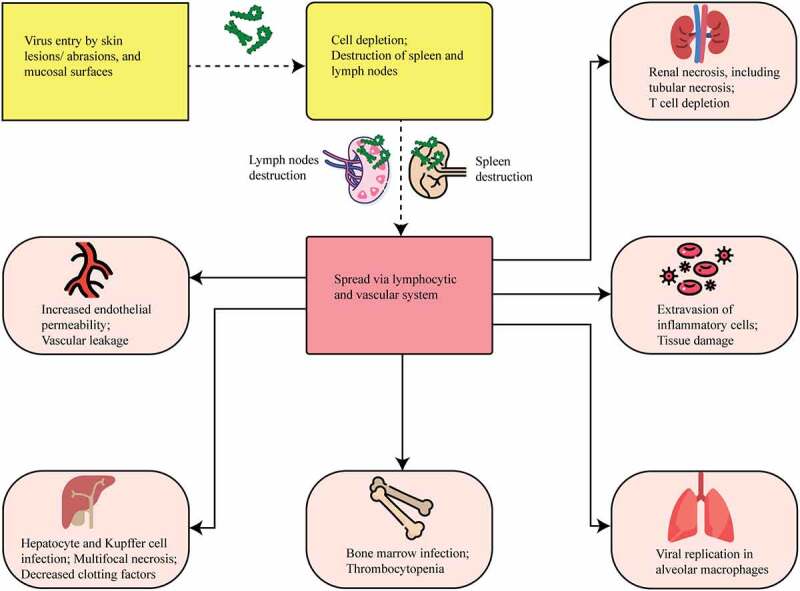
MARV entry, viral dissemination, and cellular tropism. The yellow color in the figure shows viral entry mechanisms, whereas the red color shows viral dissemination pathways. MARV enters the host and spreads throughout the lymphatic and vascular systems. The light brown color indicates the damaged cellular organelles. MARV causes necrosis in many organs, including the liver, spleen, kidneys, gastrointestinal tract, and endocardium.

The dissemination and replication of MARV in hosts facilitates the penetration and navigation of viral particles into several cells [103]. MARV gains entry to the host through mucosal surfaces, breakage and scratches of the skin, or by inoculation, and the virus gains access to tissues remote from their infection site by damaging subcellular mechanisms. The entry of MARV into the host cell includes three different phases: i) cellular attachment, ii) endocytosis, and iii) fusion. Several potential cell entry mechanisms have been proposed, which include clathrin-mediated endocytosis, macropinocytosis, and glycoprotein-facilitated receptor binding. However, these mechanisms are not mutually exclusive and apply to different parts of the entry pathway [104]. Various attachment factors, such as tyrosine kinase receptors and C-like lectins; as well as cellular receptors, such as NPC1 have been identified as possible mediators of viral entry [105]. In case of budding, the intracellular localizing of recombinant VP40 and its release in the VLP (virus-like particle) form are greatly affected by over-expression or inhibition of myosin-10 and Cdc42 proteins, which are also crucial in filopodium formation and function. Moreover, MARV VP40 can interact with the viral nucleocapsid, and provide an interface of MARV subviral particles as well as filopodia. Filopodia are closely contacted with the adjacent cells, thus usurping these structures can facilitate spreading of MARV to the neighboring cells [106]. This is why high viral titers in the blood are seen in both animals and humans infected with MARV. When viruses are released from basolateral membranes, they provide access to the underlying tissue and vasculature, leading to severe infections. Furthermore, the basolateral portion of hepatocytes as well as biliary epithelial cells were found to be responsible for MARV budding. This activity is associated with VP40 protein, which is vital for releasing infectious particles within the infected host to promote disease progression (Figure 5) [107,108].

### Cellular damage and viral tropism

MARVs are among the most devastating and virulent pathogens that affect humans. Autopsies of MARV-infected patients have revealed swelling of the heart, brain, spleen, kidneys, and lymph nodes. Also in NHPs, hemorrhages were identified in mucous membranes and soft tissues; the most severe necrotic lesions were observed in the lymph nodes, liver, spleen, testes, ovaries, gastrointestinal tract, and endocardium [67]. These organs contain a high number of reticuloendothelial cells that can migrate and spread to several organs, causing abnormal vascular permeability and the activation of clotting cascade. During the later stage of the disease, hemorrhages are seen in the gastrointestinal tract, in the pericardial, pleural, and peritoneal cavities, and in the renal tubule, associated with the fibrin deposition [59].

#### Liver

The severity of MARV’s hepatic damages is apparently greater than EBOV [59]. In MARV infections, the liver features distinctive histopathology characteristics. Studies on fatal humans and NHPs have shown that the liver represents a critical replicating organ for MARV [109]. Hepatocyte necrosis ranges from focal to widespread, with slight inflammation, which causes swelling, the degradation of liver cells and the reticuloendothelial system, mild to moderate steatosis, and upper cell hyperplasia. The elevation of liver enzymes, such as alanine amino-transferase, aspartate amino-transferase, serum glutamic pyruvic transaminase, and serum glutamic oxaloacetic transaminase, are hallmarks of MARV infection [86]. Hepatocytes may show basophilic inclusions of the cytoplasm near necrotic eosinophilic regions, consisting of viral nucleocapsid aggregates [110,111]. The asialoglycoprotein receptor has been recognized as a liver specific receptor, likely to mediate MARV infections [112]. Because the synthesis of several clotting factors occur in liver, pathologically changes in liver can potentiate abnormalities in coagulation, including DIC (disseminated intravascular coagulation), which are associated with MARV infection and increase the risk of multi-organ failure. Furthermore, hypotension and hypovolemia are consequences of adrenal gland infections, associated with the retardation of steroid-synthesizing enzymes, causing shock [113].

#### Spleen and lymph nodes

MARV infection in humans causes damage to lymphatic tissues, for example necrosis of the follicles and the medulla of lymph nodes, and red pulp of the spleen, in addition to lymphocyte depletion. Interestingly, lymphocytes are not infected by the virus, although bystander apoptosis occurrence results in lymphocyte depletion [114,115]. In NHPs, both the red and white pulp of the spleen display moderate necrosis, and lymphoid depletion in the white pulp was apparent, whereas the red pulp accumulated fibrin and cellular debris. The sinuses were covered with a small quantity of cell debris and granular material [67]. In humans, viral antigen was found in the marginal region of the red pulp and macrophages but were not detected in germinal centers, despite severe necrosis. Marburg-like inclusions were often observed in macrophages but were never detected in lymphatic cells. Fibrin and fibrinocellular debris were deposited throughout the red pulp. Sometimes virions have been observed in combination with the deposit [110,116,117].

#### Lungs

The lung alveoli often showed diffuse congestion, hemorrhage, suppurative pneumonia, and bacterial co-infection [110,116]. Small necrotic foci/micronecrosis, and fibrin were identified in alveolar macrophages, and the endothelia of alveolar capillaries were often disturbed in MARV-infected patients [110].

#### Gastrointestinal tract and small intestine

A large volume of plasma cells and monocytes exist in lymphatic organs and the mucous membranes of the stomach and intestines of NHPs [67]. In humans, the submucosa showed severe edema, including the invasion of degenerated inflammatory cells (such as neutrophils) and multiple hemorrhage foci. Autolysis of the intestine impeded cellular identification. The gastrointestinal tract showed mild focal mononuclear penetration in the lamina propria of the gastric, small intestinal and colonic mucosa. Macrophages exhibited Marburg-like inclusions, and virions were present in reticular fibrils and debris from necrotic cells. These results explain the human-to-human transmissions that can occur due to exposure to bloody stools [110].

#### Kidneys

The kidneys were swollen, pale, and hemorrhagic; tubular necrosis and parenchymal damage cause tubular dysfunction, which leads to proteinuria in MARV patients [109]. Multiple suppurative embolic foci associated with gram-negative bacteria were observed. Viral antigen was observed multifocally in glomerulus, proximal tubular epithelial cells, as well as in interstitial connective tissues near capillaries. Marburg virus-like inclusions occur in intertubular tissues macrophage and fibroblast-like cells, and some virions were observed in the glomerular capillaries, but no viral antigen was observed in the medulla [110]. Proximal tubular cells (PTCs) and mitoribosomes (mitochondrial ribosomes) were infected by MARV and induce significant changes in gene expression, leading to acute kidney injury in MARV infection via disruption of the PTC’s energy supply [118].

#### Skin

Skin and mucous membranes typically show hemorrhagic abnormalities in MHF, which is associated with skin lesions. Limited histopathological changes occur to skin tissues, including endothelial cell swelling, focal hemorrhage, necrosis, and dermal edema. Cutaneous effects appear regularly between the 2nd and 7th days after symptoms onset, and can occur during the recovery period as well [119]. Epidermal DCs, endothelial cells, connective tissue fibroblasts, and even the epithelium of sebaceous glands and sweat contain viral antigens [59].

#### Testis

A study showed that following the onset of symptoms, MARV can persist in the semen for up to 7 weeks [120]. The inclusion of viral antigens in seminiferous tubules supports the potential for sexual transmission of MARV [121]. Scrotal pain is often identified, with a few orchitis cases, and necrosis has been described in the testicles and ovaries of MHF patients [86]. Persistent testicular MARV infections among NHP survivors can lead to severe testicular injuries, including sperm cell loss and inflammatory invasion. MARV persists primarily in Sertoli cells, resulting in the breakdown of the blood–testis barrier [84]. MARV infection also induces focal orchitis, germ cell destruction, and the abundant deposition of IgG antibodies [122].

#### Bone marrow

Morphological changes to the bone marrow in MHF remain imprecisely defined. MARV antigen infects normocellular bone marrow, causing focal necrosis. However, thrombocytopenia can be observed without a concomitant reduction in platelet production in MARV cases, similar to EBOV cases [122].

#### Heart and central nervous system

Morphological myocardial injuries observed in MARV autopsy cases have not been uniform. Multiple suppurative embolic foci and lesions containing gram-negative bacteria (such as *Pseudomonas*) have been observed in the myocardium, but no viral antigen has been associated with these lesions [110]. A few MARV cases have demonstrated panencephalitis, with glial nodules and mild perivascular lymphocytic infiltration in the brain [86].

#### Endothelial cell dysfunction

Endothelial cells are one of the target cells for MARV replication because replication in endothelial cells maintains and strengthens the viremic phase. This hypothesis is supported by the observance of viral budding from the apical plasma membrane. However, basolateral budding would enable viruses to spread into the tissues even early during the infection process. Endothelial cells are responsible for the maintenance of barriers between blood and the surrounding tissue; therefore, viral replication may cause the loss of barrier function, allowing viral spread into the tissues. Endothelial cell activation and the associated secretion of inflammatory mediators can also increase vascular permeability. DIC occurs due to the increased consumption of coagulation factors, resulting in shock syndrome and hemorrhage [123,124].

## Immune evasion of MARV

The mechanisms that mediate MARV pathogenesis remain poorly established at the molecular level *in vivo* [125]. MARV VP40 plays a role in the virion as a matrix protein and has recently been shown to be involved in host innate immune antagonism through various mechanisms [126,127]. In early-stage MARV infection, no differences in B cell expression were observed in the NHP model, and changes in B cell levels did not appear until late in the infection [128]. MARV infection causes changes to the host gene expression profile within 24–48 hours after infection, and most of these changes affect genes associated with immunoregulations, coagulations, and apoptotic pathways. Moreover, the gene expression change is linked with interferon-stimulated gene (ISG) production in hepatocytes, which can act as severe antiviral suppression mediators. These findings suggest that MARV can successfully abolish interferon (IFN) reactions, including type 1 and type 2 IFN signaling, provoke an inflammatory cytokine response, and demonstrate rapid replication kinetics [129,130].

Hematological modifications have also been observed, such as early leukopenia, neutrophilia, and monocytosis, with moderate eosinophilia [86]. These changes cause immuno-suppression in MVD patients, leading to the secondary infection by bacteria [85]. Moreover, elevated levels of soluble nitric oxide and proinflammatory cytokine in the blood induce intravascular apoptosis [2]. The activity of EBOV VP35 has been shown to obstruct the development of IFN by inhibiting IFN-regulatory factor 3 (IRF­3) activation [131,132]; but this response has not yet explicitly established for MARV. Moreover, EBOV VP24 inhibits type 1 IFN signaling by restricting the nuclear importing of phosphorylated STAT1 [133]; however, MARV VP24 did not demonstrate this phenomenon. Rather, MARV VP40 did inhibit the JAK-STAT pathway by restricting the phosphorylation of both STAT1 and STAT2 [127]. However, no available data exists to describe the acquired immune responses following MHF recovery. Both IgM and IgG antibodies were observed during the DRC outbreak after symptom onset among MARV survivors, indicating the development of MARV-specific antibodies [134].

## Pathophysiology of Marburg virus

MARV usually penetrates the body through cracked skin, causing damage to multiple cell types and organs, resulting in Marburg hemorrhagic fever (MHF) [135]. The most severe clinical features of MVD include inappropriate fluid distribution, coagulation complications, shock, and multi-organ failures. MARV mainly infects macrophages, monocytes, Kupffer cells, and DCs, according to evidence from MARV-infected monkeys [115]. MARV targets mononuclear phagocytic cells, for instance monocytes and macrophages, triggering the cellular activation and permitting damage to secondary targets, such as endothelial cells [10,11,67]. Moreover, the activation of macrophages and monocytes releases cytokines and pro-inflammatory mediators, resulting in the progression of shock, which is a primary cause of death in MVD [10,136].

### MARV pathophysiology in humans

Limited comprehensive clinical studies exist for MARV due to the rural conditions as well as severe occurrences of most of the MARV outbreaks in Africa, and the compilation of laboratory and pathological evidence from patients has traditionally been inadequate. Clinical findings to date emerge from information linked to the first epidemic in Germany, with the South African outbreak, and smaller outbreaks in other areas of Africa [137]. Experiments on cultured cells of the MARV patient indicated substantial adaptive reactions against MARV by increasing immune cells, in beginning. In addition, immunoglobulin G (IgG) reaction to the viral NP and GP was found while investigating the serum sample of patients, and 2 patients had notable neutralizing titer of the antibodies. Gradually, neutralizing antibody titer declined, and this declination started 21 months post-infection, and diminished lower than detecting limit 27 months post-infection [138].

**Figure 6.**
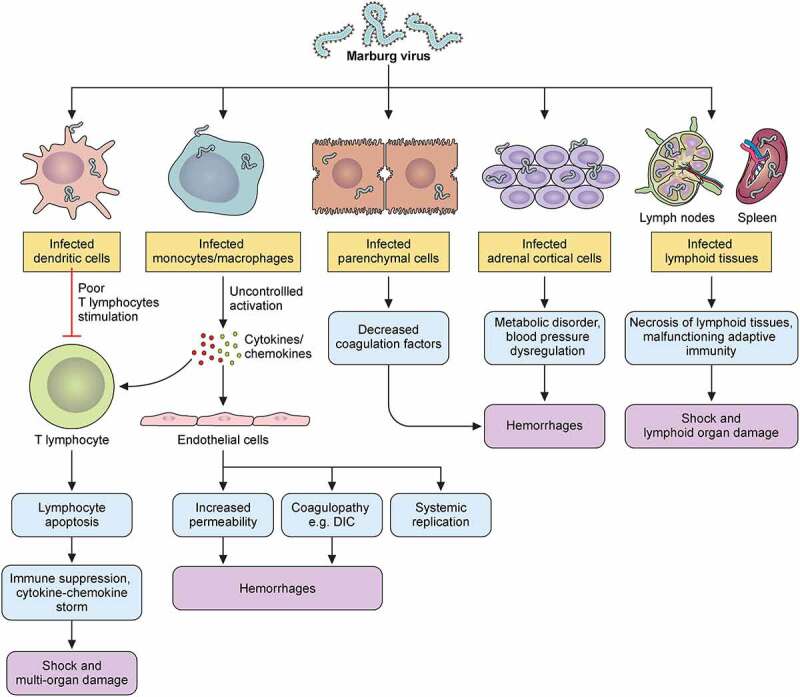
MARV hemorrhagic fever pathophysiology model in humans. Marburg virus primarily targets dendritic cells, monocytes, parenchyma cells at a liver, adrenocortical cells, and several lymphoid tissues. Infection of dendritic cells leads to poor stimulating condition of T lymphocyte that causes lymphocyte apoptotic condition. Due of this, body’s immunity is suppressed and cytokines/chemokines number is increased, which leads to shock as well as multiorgan damaging occurrence. Macrophage or monocyte infection leads to uncontrolled cytokines/chemokines activation, and they continue the damaging of T lymphocyte and endothelial cell. Endothelial cell infection causes increase of blood vessels permeability and DIC (disseminated intra-vascular coagulopathy), while both occurrences lead to hemorrhages. Systemic replication can also occur because of this infection in endothelial cells. Parenchymal cell infection occurrences in liver can decrease coagulation factors, and these occurrences can cause hemorrhages later on. Adrenocortical cells of adrenal gland infecting occurrence by MARV can lead to disorders in the metabolism and dysregulated blood pressure; and hemorrhage occurs at a later stage due to these infections. MARV infection the on lymphoid tissues of lymphatic system, especially lymph nodes and spleen infections lead to tissue necrosis and malfunctioning adaptive immunity. Shock and lymphoid organ damage can occur in the later stage.

At organ level, in patients with MARV infection, it seems that MARV mainly targets the adrenal glands and the liver (Figure 6), as well as lymphoid tissues for replication [139]. Autopsies performed on RAVV infected patients, a close-relative of MARV, indicated multiple malignant effects, including the swelling of the kidneys, heart, brain, and lymphoid tissues, in addition to hemorrhages of the soft tissues and mucosa [67]. The hepato-tropism of MARV was discovered in an *in vitro* analysis, suggesting that the hepatocyte receptor asialoglycoprotein may worsen MARV infections [112]. The necrosis of parenchymal cells in the liver destroys the reticuloendothelial system, resulting in increased liver enzyme levels in association with MARV infection [96,140]. The infection of other organs can result in various symptoms, including proteinuria due to malfunction of kidney [134], heart and lungs hemorrhages [1], scrotum pain, and necrosis of the testicles and ovaries [114].

At the cellular level, macrophage, and dendritic cell (DC) are the primary targets for MARV entrance [110,141]. These findings were supported by the identification of virions and antigens associated with the virus using immunohistochemical and electron microscopy experiments during the 1987 Kenyan outbreak [110], as well as by the observation of viral infections in the macrophages of macaques based on the results of peripheral smear of blood mono-nuclear cell populations from flow cytometry study [128]. The infection of DCs causes the paralysis of inherent immune response, and the abnormalities of lymphocytes stimulation [125]. The macrophage infections promote the production of proinflammatory cytokines including TNF-α (tumor necrosis factor-alpha), which can cause bystander apoptosis in lymphocyte populations, leading to immunosuppression and lymphopenia (Figure 6). By stimulating monocytes, infected macrophages help in the dissemination of MARV, which induces the development of cytokines and chemokines [142,143]. Although the activation is not associated with MARV replication, this process contributes significantly to viral spread and delineates the pantropic nature of MARV and annihilation of focal tissues [10]. Furthermore, modifications in vascular permeability are induced by interleukin (IL)-6 and TNF-α, which are both formed by macrophages [144]. Furthermore, tissue factors production by infected macrophage leads to the abnormal coagulations, such as DIC, and this is additionally supported by liver hepatocyte cells infection and causing decreased coagulating factor synthesis from liver (Figure 6) [145]. Infections on adrenocortical cells cause hypotension as well as metabolism disturbance, and these can cause multiorgan failure and shock, together with immunosuppression and coagulopathy (Figure 6) [146].

### MARV pathophysiology in animals

MARV pathophysiology has been observed in animal models, including guinea pigs, mice, hamsters, baboon [147,148], and certain species of NHPs, such as rhesus macaques, common marmosets, cynomolgus macaques, squirrel monkeys, and African green monkeys [149,150]. MARV has been shown to infect Kupffer and monocytes cells, as well as DCs and macrophages, in animal models. The reduction of NK (natural killer) cells, and CD4 and CD8 T lymphocyte cells generally occur throughout the viral infection time because of the death of these cells through apoptosis [67,151].

In the MARV-infected humanized mouse model, Marburg virus manifests malignancy in a greater range. MARV primarily infects macrophages, B cells, T cells, NK cells, and phagocytes in these mice [152]. In non-adapted wild type MARV-Ravn infected mice models, virus infection causes acute illness, faded and enlarged livers, featuring necrosis, enlarged spleens, and reduced lymphocytic cell populations [153], while mouse-adapted MARV-Ravn models are accompanied by several symptoms, such as reduced physical activity, weight loss, rumpled fur 3 to 4 days post-infection, and death within 5 to 7 days [154].

Guinea pig shows mild feverish manifestation when infected by host-adapted variant of MARV. However, consecutive passaging the guinea pigs with MARV can cause malignant conditions among the animals. In fact, one of the first MARV guinea pig model studies showed that 100% of the MARV-inoculated guinea pigs die 7–9 days post-infection after 8 passages by whole blood [155]. This feature was further used to generate and characterize a guinea pig model against MARV Angola strain. In the experiment of MARV Angola strain on guinea pigs, it was substantiated that MARV infections can cause lymphocytopenia, thrombocytopenia, as well as greater infecting occurrences in kidney cells, liver cells, spleens, and lungs [156]. The viral pathophysiology of MARV in guinea pigs appears to resemble those observed in mice and humans. Furthermore, vaccination studies have been widely performed in guinea pigs, and one study testing the efficiency of trivalent rVSV vaccine vectors demonstrated that 100% protection is possible when MARV infection occurs in them [157].

Syrian hamster models usually show no manifestations when infected with wild-type MARV strains. But STAT2 null immunodeficient hamster models show infections of liver and spleen, neutrophil leukocytosis, and an increase of proinflammatory cytokine [158]. Besides, another study confirmed that hamster-adapted variant of MARV in Syrian golden hamster models manifest similar pathophysiological response to MARV infection as those observed in human and NHP, including similar clinical symptoms, such as rashes, hemorrhages, coagulopathies, and malfunctioned immune response [159].

Among all tested NHP models, rhesus and cynomolgus macaques are the best-characterized as well as widely used in MARV research due to their accurate recapitulation of human pathophysiology [160]. MARV infection on NHPs showed that lymphocytosis is prevalent in the initial stages [161], and leukocytosis and thrombocytopenia is seen within 5 to 6 days after infection because of the increased neutrophil levels. MARV then infects adrenal glands, hepatocytes, as well as lymphoid tissues. During the final stages, the virus infects the endothelia of several organ tissues. The viral antigens can be found in several organs, including the kidneys, liver, spleen and adrenal glands. Typically, diarrhea, anorexia, fever, rash, and hemorrhage may manifest after 2 to 6 days of infection, and viremia arises on the 3rd day. The highest titer is typically observed after 8 days of infection [115].

In ferrets, an experiment recently demonstrated that MARV neither causes disease nor causes viremia in them [162]. Although the serology reports for MARV infection were positive and these ferrets produced neutralizing antibodies, they did not develop clinical disease [163]. Additional studies on ferrets may contribute to our comprehension of the various mechanisms that contribute to differences in pathophysiology, hence improving our understanding of the disease.

## Management approaches for Marburg virus

No established management approach for MARV currently exists. Nevertheless, a popular treatment approach is the utilization of palliatives for pain alleviating purpose. Moreover, supportive treatment is often offered, including blood volume administration and electrolyte balance [164]. Although there are currently no clinically approved vaccines or therapeutics to prevent or treat MVD, there are certainly established and effective approaches that can be used to manage outbreaks and cases.

Several supportive methods and therapies were used pre-clinically and clinically in the past, as well as several new approaches that have been studied in animal models [2,12,165–167]. In supportive therapies, antibiotic was recommended in almost each of the outbreaks; yet numerous improved supportive therapeutic approaches were established in every outbreak. At the time of the first MARV occurrence in 1967, a variety of supportive approaches rather than only antibiotics were used, including cardiac glycosides, antipyretics, steroids, electrolytes, convalescent serum, and fluids supplement [85,168]. Clinicians introduced the use of parenteral fluids, analgesics, prophylactic heparins, and plasma of Lassa fever patients to the infected humans during the second outbreak [46,169]. Anti-malarial drugs and antibiotics were used in the 1980 outbreak of Kenya [47]. Moreover, in the 1987 Kenya outbreak, heparins, steroids, antibiotics, and plasma were utilized, and concurrently dialysis was proceeded [48]. In the 1990 Russian outbreak, extracorporeal hemosorbents were used, in addition to hemodialysis [63]. In addition to antibiotics and anti-malarials, the next epidemic in the DRC in 1998–2000 had the implementation of modern acetaminophens, antiemetics, antacids, and intravenous fluids [49]. During the 2004–2005 Angolan epidemic, no treatment was provided to patients initially, but antimalarials, antibiotics, analgesics, antiemetics, sedatives, and cimetidine were provided later, as well as oral rehydration was administered concurrently during the first 3 months and intra-venous fluid were given after 3 months [170–172]. Although the information was unavailable regarding the MARV management of Uganda outbreak initially in 2007 but, some supportive managements were provided during the 2008 Uganda-origin outbreak of MARV, including blood transfusion, malaria prophylaxis, antiemetics, cholecystectomies, and antibiotic for the case that transferred to the USA [52]. Moreover, intravenous fluid, plasma, hemofiltration, and hypertonic saline were provided to the case that transferred to the Netherlands [166]. Newly, an innovative approach of remote controlled and pressure guided fluid supplementation has been proposed for the efficacious management of filovirus infected patients [173].

Modern researches have investigated various drugs for MARV infection. A recent study of remdesivir against Marburg virus showed that it has therapeutic efficacies in cynomolgus macaque models, which makes it an important drug to be assessed further. The study showed that it had therapeutic efficacy when given at 4 or 5 days post inoculation once daily in vehicle, for 12 days with 5 mg/kg dose, or 10 mg/kg loading dose followed by 5 mg/kg dose [174]. Another study showed that cholesterol conjugated fusion inhibitors are active *in vitro* against Marburg virus [175]. Recently, 4-(aminomethyl) benzamide has also been substantiated to be a potent entry inhibitor of MARV infection [176]. Furthermore, researchers demonstrated 33 hit compounds *in vitro* against MARV with their several pharmacological potentials [177]. Some small molecules, such as BCX4430, favipiravir, aloperine etc. have also been showed to be effective against MARV infection [178]. Again, an inhibitor compound FC -10696 has recently been discovered to inhibit the egress of MARV [179]. Also, AVI-7288 has been indicated to show potentials as a post-exposure prophylaxis against MARV [180].

Numerous experiments were conducted on rodent and NHP models for testing vaccine efficacies against MARV. To date, some vaccines have been trialed for human use [78,181,182]. Among them, cAd3 vaccine, also known as chimpanzee adenovirus serotype 3 vector, encoded with wild type GP from MARV, is in phase 1 clinical trial for human use [183]. The MVA-BN-Filo vaccine, also known as the modified vaccinia Ankara vector vaccine, is encoded with GP from Ebola, Sudan, MARV, and NP from Tai Forest virus. It has been planned for phase 2/3 trials after the successful completion of phase 1 trial [78,184]. MARV DNA plasmid vaccine, which is a MARV DNA plasmid that expresses GP from MARV Angola [185], as well as from MARV Sudan [186]. Both of them completed phase 1 clinical trial [78]. rVSV-MARV-GP vaccine is recombinant vesicular stomatitis virus vector for MARV GP, that has been studied in NHP models, but human trial is not conducted yet [78,187,188]. In addition, VLP vaccine or virus-like particles with GP vaccines are yet to be trialed in humans, as NHP studies of VLP vaccine have been completed [189,190]. In recent times, single vial trivalent vaccines have been invented, which showed high antibody levels in mouse and NHP models. This vaccine might help in easier distributing and administrating procedures of vaccines in rural and poor areas [191]. Hence recombinant subunit vaccines platform should be allowed to develop safer and efficient multivalent vaccine candidates for protection against MARV [192].

The experimental approaches have substantiated the efficacies of numerous treatments, which include anti-viral treatment, pre- and post-exposure vaccine treatment, and treatment with IFNs; these are displayed in the Table 6. However, Experimental approaches are still being processed in animal models as well as in humans to determine the efficacy of treatments and vaccines. A very recent study substantiated that combination therapy, especially usage of monoclonal antibody (MR186-YTE) with remdesivir, can significantly protect macaques from MARV infection. The study showed that monoclonal antibodies alone can give 100% protection, and 80% protection in NHPs when used together with remdesivir 5 days post-infection [193]. Therefore, further investigation of monoclonal antibodies and combination therapies, as well as their human implementation can be another possible dimension of MVD management.

**Table 6.** Evaluation of Marburg virus treatment in NHP models

Treatment types	Animal models	Compounds used	MARV Strain	First dose after infection	Dose	Dose numbers	Survival rate	Ref.
Antibody based treatment	Rhesus macaque	MR191-N	Angola	4 days	50 mg/kg	2	100%	[194]
				5 days		2	80%	
			Ravn	5 days		2	100%	
	Rhesus macaque	Purified Immunoglobin G	Ci67	15–30 minutes	100 mg/kg	3	100%	[195]
				2 days		3	100%	
Antiviral treatment	Cynomolgus macaque	BCX4430	Musoke	1 hour	15 mg/kg	30	83%	[196]
				1 day		28	100%	
				2 days		26	100%	
	Rhesus macaque	siRNA NP	Angola	1–4 days	0.5 mg/kg	7	100%	[197,198]
				5 days		7	50%	
			Ravn	3 days	0.5 mg/kg	7	100%	[198]
				6 days		7	100%	
	Rhesus macaque	PMOplus (pool)	Musoke	30–60 minutes	40 mg/kg	14	100%	[199]
	Cynomolgus macaque	PMOplus (NP)	Musoke	1 day	15 mg/kg	14	83%	[200]
				2 days		14	100%	
				4 days		14	83%	
	Cynomolgus macaque	GS-5734	Angola	4 or 5 days	10 mg/kg loading dose, then 5 mg/kg	12	83%	[174]
				5 days	5 mg/kg	12	50%	
Post-exposure vaccines	Rhesus macaque	rVSV-MARV	Musoke	20–30 minutes	10^7^ PFU	1	100%	[201]
	Rhesus macaque	rVSV-MARV	Musoke	1 day	2 × 10^7^ PFU	1	83%	[202]
				2 days		1	33%	
Pre-exposure vaccines	Cynomolgus macaque	rVSV-MARV	Musoke, Popp	-	10^7^ PFU	1	100%	[203]
	Cynomolgus macaque	rVSV-MARV	Musoke, Angola, Ravn	-	2 × 10^7^ PFU	1	100%	[204]
	Cynomolgus macaque	rVSV-MARV	Angola	-	2 × 10^7^ PFU	1	100%	[205]
	Cynomolgus macaque	rVSV-ZEBOV + rVSV-SEBOV + rVSV-MARV	Musoke	-	10^7^ PFU	1	100%	[206]
	Cynomolgus macaque	VLPs + QS-21 adjuvant	Musoke, Ci67, Ravn	-	1 mg VLPs +.1 ml QS-21	3	100%	[190]
	Cynomolgus macaque	mVLPs + adjuvant	Musoke	-	3 mg VLPs +.1 mg QS-21 or .5 mg/kg polyI:C	3	100%	[189]
	Cynomolgus macaque	DNA MARV GP	Musoke	-	20 µg	3	67%	[207]
	Cynomolgus macaque	DNA MARV GP	Angola	-	4 mg	4	100%	[208]
	Cynomolgus macaque	DNA MARV GP + DNA RAVV GP + DNA EBOV GP + DNA SUDV GP	Musoke	-	500 µg individual or 2 mg total	3	100%	[209]
	Cynomolgus macaque	DNA MARV GP + rAD5 MARV GP	Angola	-	4 mg DNA GP + 10^11^ PU rAD5 GP	4	100%	[208]
	Cynomolgus macaque	VRP-MARV GP and/or NP	Musoke	-	10 × 10^6^ FFU	3	67–100%	[210]
	Cynomolgus macaque	CAdVax-panFilo	Musoke, Ci67, Ravn	-	4 × 10^10^ PFU	2	100%	[211]
	Cynomolgus macaque	rAD5 MARV GP	Angola	-	10^11^ PU	1	100%	[208]
	Rhesus macaque	Inactivated MARV	Popp	-	7 µg	2	50%	[212]
Interferons	Rhesus macaque	rNAPc2	Angola	10 minutes	30 µg/kg	15	17%	[161]
	Rhesus macaque	IFNβ	Musoke	1 hour	35 µg/kg	15	33%	[213]

## Conclusion and future prospects

Rural and poor conditions, along with sporadic outbreaks in Africa, are principal reasons for unsatisfactory clinical findings of the Marburg virus. Although the African fruit bat has been identified as a potential natural reservoir for MARV and most of the outbreaks have been caused by spillover into human population from an animal reservoir, human-to-human transmission mediated by the care providers or health professionals who work in hospitals also played great role in the growth of MARV outbreaks. These outbreaks demonstrated that MARV could be malignant and able to cause severe outbreaks if not taken proper steps immediately. Although MARV strains originate only in African regions, the outbreak history suggests that other continents could also be at risk. Recent outbreaks have indicated the necessity of additional research exploring the pathophysiology and pathogenicity of MARV. Current data remain based on earlier studies of infected patients. In response to the intensity of previous outbreaks, epidemiologists have suggested MARV as a threat to global public health. Therefore, novel studies examining MARV remain crucial to provide unambiguous guidelines for the therapeutic management of patients, and for the development of vaccines. These steps could be used to minimize future outbreaks and reduces the CFRs. Vaccine studies have been ongoing for the past few years, and the virus is being studied on a variety of animal models, including NHPs, mouse models, guinea pigs, and hamsters. Vaccine studies should be perpetuated until the licensure and distribution of vaccines in humans occurs.

## Data Availability

The authors confirm that the data supporting the findings of this study are available within the article [and/or] presented in the table and figures.
